# Encapsulation of Dexamethasone into mRNA–Lipid Nanoparticles Is a Promising Approach for the Development of Liver-Targeted Anti-Inflammatory Therapies

**DOI:** 10.3390/ijms252011254

**Published:** 2024-10-19

**Authors:** Ignacio Rivero Berti, Rocío Celeste Gambaro, María José Limeres, Cristián Huck-Iriart, Malin Svensson, Silvia Fraude-El Ghazi, Leah Pretsch, Shutian Si, Ingo Lieberwirth, Katharina Landfester, Maximiliano Luis Cacicedo, Germán Abel Islan, Stephan Gehring

**Affiliations:** 1 Children’s Hospital, University Medical Center of the Johannes Gutenberg University, Langenbeckstr. 1, 55131 Mainz, Germany; ignaciob@uni-mainz.de (I.R.B.); gambaror@uni-mainz.de (R.C.G.); mj.limeres@uni-mainz.de (M.J.L.); malin.svensson@uni-mainz.de (M.S.); fraudesi@uni-mainz.de (S.F.-E.G.); pretsch@uni-mainz.de (L.P.); maximilianocacicedo@gmail.com (M.L.C.); 2 CINDEFI—Centro de Investigación y Desarrollo en Fermentaciones Industriales, Laboratorio de Nanobiomateriales, Facultad de Ciencias Exactas, Universidad Nacional de La Plata (UNLP)-Consejo Nacional de Investigaciones Científicas y Técnicas (CONICET), La Plata 1900, Argentina; 3 ALBA Synchrotron Light Source, Carrer de la Llum 2–26, Cerdanyola del Vallès, 08290 Barcelona, Spain; chuck@cells.es; 4 Max Planck Institute for Polymer Research, Department of Physical Chemistry of Polymers, Ackermannweg 10, 55128 Mainz, Germany; sis2@mpip-mainz.mpg.de (S.S.); lieberw@mpip-mainz.mpg.de (I.L.); landfest@uni-mainz.de (K.L.)

**Keywords:** lipid nanoparticles, mRNA delivery, dexamethasone, cytokines, dendritic cells, hPBMCs, in vivo biodistribution, anti-inflammatory

## Abstract

The objective of this study was to develop two lipid nanoparticle (LNP) formulations capable of efficiently expressing a reporter mRNA while co-delivering the anti-inflammatory drug dexamethasone (DX) to reduce inflammatory side effects in protein replacement therapies. Two types of LNPs were developed, in which 25% of cholesterol was replaced by DX. These LNPs contained either 1,2-distearoyl-sn-glycero-3-phosphocholine (DSPC) or 1,2-dioleoyl-sn-glycero-3-phosphoethanolamine (DOPE) as a helper lipid. The resulting LNPs exhibited high stability, homogeneity, and near-neutral Zeta potentials. SAXS experiments confirmed DX incorporation into the LNP core, with slow in vitro DX release observed over 48 h. The LNPs achieved high mRNA encapsulation efficiency (95–100%) and effectively transfected HepG2 cells, dendritic cells, and hPBMCs. While LNPs increased cytokine release (IL-1β, TNF-α, MCP-1), LNPs-DX significantly reduced cytokine levels, demonstrating enhanced anti-inflammatory properties while maintaining mRNA expression levels. In vivo biodistribution showed predominant liver localization post-intramuscular injection, regardless of the DSPC or DOPE composition. LNPs co-loaded with mRNA and DX are promising candidates for continuous protein replacement. Due to their ability to reduce treatment-related inflammation while maintaining significant mRNA expression levels, these LNPs are perfectly suited for the treatment of liver-related metabolic diseases.

## 1. Introduction

In the landscape of mRNA delivery, the pursuit of optimal formulations is a priority, aligning with the broader goal of enhancing therapeutic efficacy while minimizing side effects [1]. The licensed vaccines from Moderna and Pfizer/BioNTech against SARS-CoV-2 have demonstrated the potential of LNPs as effective carriers for mRNA [2]. However, they have also been associated with side effects ranging from mild reactogenicity to rare but severe conditions [3]. Reactogenicity associated with LNPs is usually more pronounced following the administration of the second vaccine dose and includes fever, muscle aches, fatigue, and headache.

The induction of pro-inflammatory cytokines by LNPs can enhance immune responses and improve vaccine efficacy. However, it has also been shown to potentially lead to immunological adverse effects. Furthermore, the complement system is activated when polyethylene glycol (PEG), an LNP component, interacts with pre-existing anti-PEG antibodies in the body [4,5].

Modification of the architecture of ionizable lipids, phospholipids, cholesterol, and PEG lipids represents a promising avenue for the development of optimized carriers that achieve high mRNA expression and low reactogenicity [6]. However, the complex interactions between LNP components and the immune system remain a significant challenge [7].

Cholesterol is typically one of the four major lipidic components required to synthesize LNPs, typically constituting between 35 and 45% of the total lipidic structure. Cholesterol plays two primary roles: (1) it increases the stability of the LNP, and (2) it assists in mRNA transfection. LNPs can be modified by using alternative molecules structurally related to cholesterol [8]. A wide variety of cholesterol-like molecules, such as phytosterols, can be found in nature and may be relevant for improving mRNA transfection [9]. Other researchers have proposed using corticosteroids to modify the LNP structure [1,10]. This approach offers the advantage of generating LNPs with anti-inflammatory properties. Specifically, dexamethasone (DX) is a potent synthetic glucocorticoid with anti-inflammatory and immunosuppressive activities. Its chemical structure presents some similarities with cholesterol, making it a suitable candidate to replace cholesterol in LNP formulations [10].

Several reports suggest that DX plays a key role in reducing the release of pro-inflammatory cytokines by immune cells, which might have a beneficial effect on mRNA translation [1,10]. Other approaches have attempted to use a DX prodrug in LNPs to suppress cytokine production after intravenous administration [11]. Furthermore, co-administration of DX has been proposed as a potential clinical strategy to reduce the inflammatory effects of liposome treatments, particularly by increasing the safety profile of siRNA-based drugs in multiple tissues [12].

However, the pharmacokinetics of DX-loaded LNPs are not well understood, considering the potential side effects of systemic glucocorticoid administration. DX can produce differential effects on the thymus and spleen by altering programmed cell death, modifying lymphocyte subgroups and T cell activation, and causing thymus reduction [13].

The present study investigates the role of DX co-loaded into LNPs and the effects of this LNP formulation on mRNA expression. A rational study was conducted on the physicochemical properties, encapsulation, and pharmacokinetics of DX released from different LNP formulations, which was not previously explored in other publications. The incorporation of DX into stable LNPs was confirmed by SAXS studies. We examined the biological effects on eukaryotic cells, focusing on the concomitant release of pro-inflammatory cytokines. Several reports have studied the effects of similar DX-LNP systems in mouse models [1,10]. However, our work additionally investigates the effects of DX-mRNA co-loaded LNPs on human primary peripheral blood mononuclear cells (hPBMCs) and assesses hemotoxicity in human blood samples. This research provides valuable insights into the ability of DX-LNPs to deliver mRNA and the potential to treat inflammatory diseases. Furthermore, it potentially offers an optimized approach for mRNA-based protein replacement therapies, as previously reported for tyrosinemia and phenylketonuria [14,15]. Repeated dosing in these therapies could potentially lead to reactogenicity issues due to immune responses against the encoded proteins and/or the LNP components, which could compromise the efficacy of the therapy.

## 2. Results

### 2.1. Effects of Increasing DX Concentrations on LNP Characteristics and Biological Properties

LNPs were formulated by mixing an aqueous solution containing the mRNA constructs (EGFP or Luc) with an ethanolic phase containing the lipids using the NanoAssemblr^®^ microfluidic platform from Precision NanoSystems Inc.(Vancouver, BC, Canada) [6]. The theoretical composition of the LNPs is estimated based on the precise mass of individual components (ALC-0315, DSPC, or DOPE; cholesterol or DX; ALC-0159) added to the ethanolic phase. Since LNPs were then produced using the nanoprecipitation method, all initial components were expected to remain encapsulated, and the molar ratios were assumed to match those of the lipid mixture.

**Figure 1:**
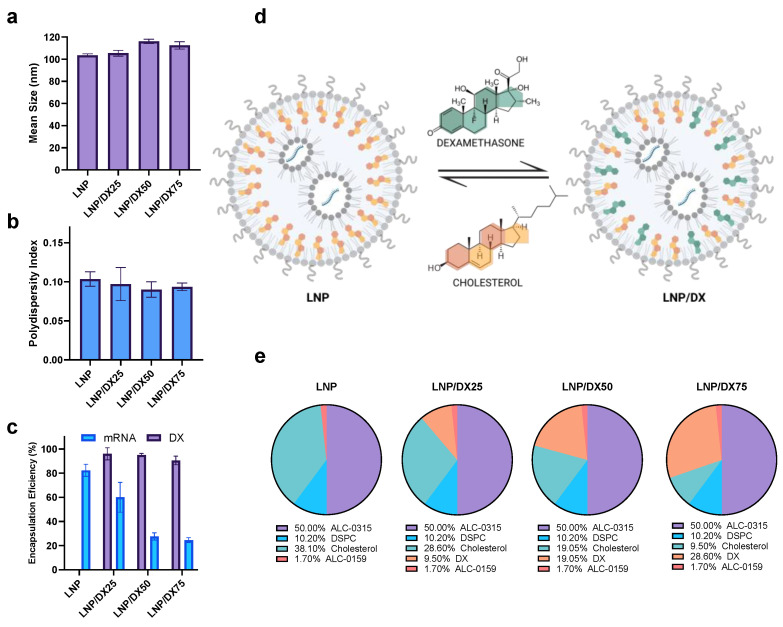
Screening of LNPs with different compositions by increasing DX concentrations into LNPs after cholesterol replacement prepared with the microfluidic system NanoAssemblr^®^ Spark™. The effect of DX replacement on mean size (**a**) and PDI (**b**) by DLS, as well as the encapsulation efficiency (**c**) of the cargo molecules (EGFP mRNA and DX), were evaluated. Scheme of cholesterol replacement by structurally related analogs (DX: dexamethasone) into the LNP architecture (created with BioRender.com) (**d**). Composition of the different LNP formulations in mol (%) with increasing concentrations of DX (**e**).

As a first approach, LNPs composed of ALC-0315, DSPC, cholesterol, and ALC-0159 were synthesized using the NanoAssemblr^®^ Spark™ (Vancouver, BC, Canada) microfluidic system. In the lipid mixture, cholesterol was partially replaced with increasing DX concentrations, ranging from 25% to 75% (Figure 1). These two compounds, classified as steroids, have similar chemical structures and molecular weights, with DX at 392.5 and cholesterol at 386.7 KDa [16]. Due to its hydrophobic nature (logP of 7.02 and 1.87 for cholesterol and DX, respectively), DX is well-suited for LNP formulation, facilitating co-assembly with other lipids through hydrophobic interactions [10].

The effect of this modification on the structural properties of the LNPs was studied in order to select the optimal conditions for mRNA encapsulation while maintaining DX incorporation into the lipid structure. The mean size of the LNPs showed a minimal increase after 50% and 75% DX replacement, from the initial 103 nm to 116 nm and 113 nm, respectively (*p* < 0.05). The PDI values remained below 0.1, indicating good homogeneity in the formulations. Notably, DX encapsulation efficiency remains consistently around 90% in all tested formulations. However, a progressive decline in mRNA encapsulation was observed. While the initial LNPs exhibited an mRNA encapsulation efficiency (EE%) of approximately 82%, a decrease to 60%, 28%, and 25% was observed for LNPs with 25%, 50%, and 75% replacement of cholesterol, respectively.

**Figure 2:**
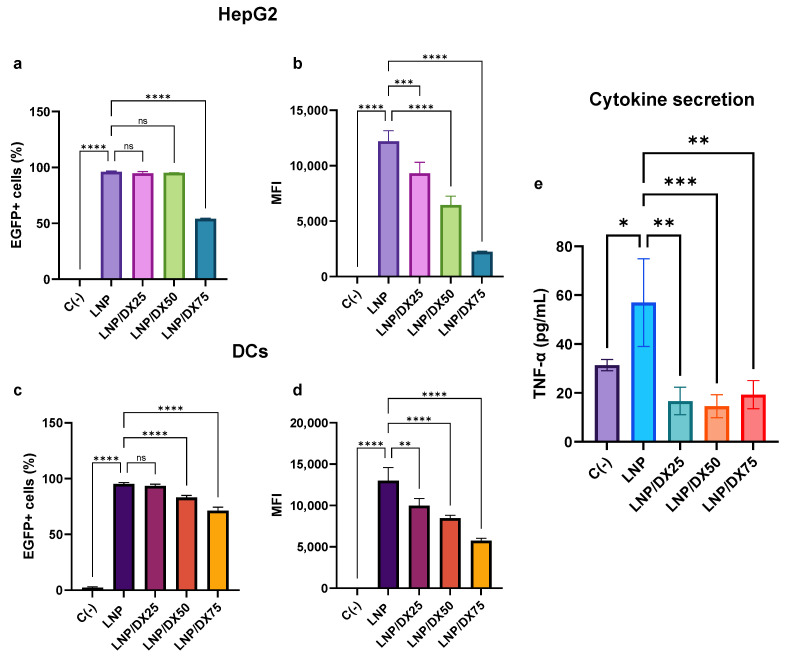
Transfection efficiency of the LNP/DX formulations in HepG2 cells (**a**,**b**) and mouse dendritic cells DC 2.4 (**c**,**d**) with a total mRNA of 1 µg/mL for 24 h. Stimulation with LNP and cytokines secretion by DCs was also determined (**e**). Results represent the mean (n = 3) ± SD. Abbreviations: C−: untreated cells. One-way ANOVA followed by Fisher’s LSD test was used to compare between groups: *p* < 0.05 (*), *p* < 0.01 (**), *p* < 0.001 (***), *p* < 0.0001 (****), and ns (not significant; *p* > 0.05).

As a second quality control, the ability of the different LNPs to transfect eukaryotic cells was assessed. The HepG2 and DC 2.4 cell lines were selected as reference models to evaluate the transfection efficiency of the LNPs (Figure 2). Cells were transfected with a total mRNA concentration of 1 µg/mL to maintain the increasing concentrations of DX.

Approximately 95% of HepG2 cells showed positive transfection with EGFP-encoding mRNA loaded into LNP, LNP/DX25, and LNP/DX50 (Figure 2a,b). In contrast, only 54% of EGFP-positive cells were observed after treatment with the highest dose of DX (LNP/DX75). In terms of mean fluorescence intensity (MFI), there was a nearly linear decrease in mRNA expression as the amount of DX in the LNP increased. This could be explained by considering the EE of the different formulations, which decreased as much as the DX replacement was raised. A similar trend was observed in the case of DC 2.4 cells, where no significant differences (*p* > 0.05) were found in the total percentage of transfected cells between LNP and LNP/DX25. Conversely, a 10% and 25% decrease in EGFP signal was observed with LNP/DX50 and LNP/DX75, respectively (Figure 2c,d). In addition, the mRNA expressed per cell (MFI) showed a linear decrease as the proportion of DX in the LNP increased. The viability of both cell types was not affected by LNP treatments (Figure S1).

To evaluate the anti-inflammatory effects of DX and to verify whether these properties were maintained after its encapsulation in LNPs, the release of TNF-α from DC 2.4 cells was quantified (Figure 2e). The results showed that LNP treatment for 24 h induced TNF-α release at levels at least twice that of the basal production. However, TNF-α release decreased approximately fourfold when cells were treated with all DX-containing LNPs. These levels were in the order of the negative (basal) control, clearly indicating that DX acts as an anti-inflammatory molecule, even when it is encapsulated.

Based on these screening data, the LNP formulation with a 25% DX/cholesterol replacement was selected for the subsequent experiments. LNP/DX25 exhibited the highest percentage of transfected cells and the highest mRNA expression while also demonstrating the ability to reduce the production of inflammatory cytokines.

### 2.2. Preparation of DX-Loaded LNPs Composed of DSPC and DOPE Using the NanoAssemblr^®^ Ignite™

**Figure 3:**
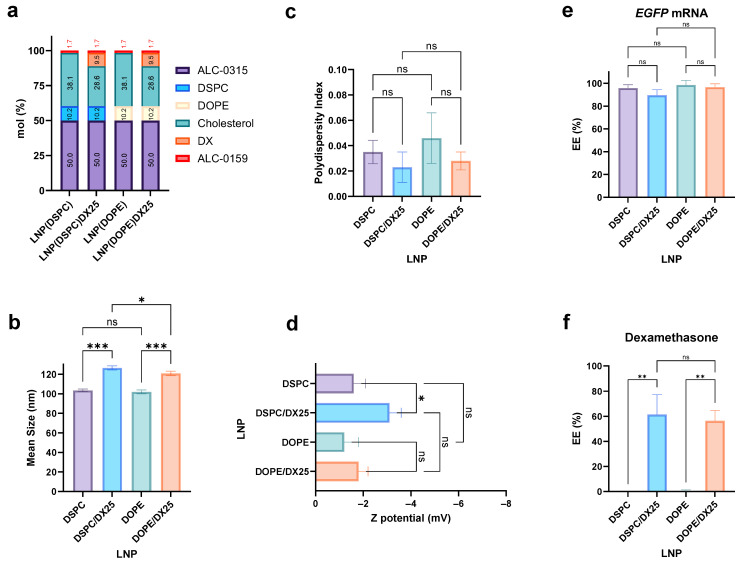
Screening of DX concentrations into LNPs with NanoAssemblr^®^ Ignite™. Composition of the different formulation with DSPC or DOPE helper lipids (**a**). The mean size (**b**), PDI (**c**), and Z potential (**d**) of the LNPs were determined. The encapsulation efficiency of the reporter EGFP mRNA (**e**) and DX (**f**) was calculated. The results represent the mean (n = 3) ± SD. One-way ANOVA followed by Fisher’s LSD test was used to compare between groups: *p* < 0.05 (*), *p* < 0.01 (**), *p* < 0.001 (***), and ns (not significant; *p* > 0.05).

In the next step, the 25% DX/cholesterol replacement formulations were prepared using the NanoAssemblr^®^ Ignite™ (Vancouver, BC, Canada) microfluidic device. Considering the effect of helper lipids in LNP architecture, two formulations by modifying the phospholipids (DSPC and DOPE) were proposed (Figure 3a). It was reported that LNPs formulated with DOPE exhibited stronger interactions with ApoE, leading to higher accumulation in the liver of injected mice. In contrast, LNPs formulated with DSPC showed weaker interactions with ApoE and were more predominantly accumulated in the spleens of the injected mice [17].

Stable LNPs were obtained with sizes around 100 nm for LNP and approximately 120 nm for DX-loaded LNPs (Figure 3b). The LNPs demonstrated high homogeneity indices, with PDI values below 0.05 (Figure 3c). The Zeta potential of all formulations remained close to neutrality, ranging from −1 to −3 mV (Figure 3d). The encapsulation efficiency (EE) of mRNA was around 90% for all formulations, indicating that DX did not affect the EE (Figure 3e). In addition, approximately 60% of DX was successfully encapsulated in both formulations (Figure 3f). At this point, no significant differences were observed (*p* > 0.05) in the studied physicochemical properties of LNPs by replacing DSPC with DOPE.

### 2.3. Physicochemical and Pharmacodynamic Characterization of DX-Loaded LNPs

**Figure 4:**
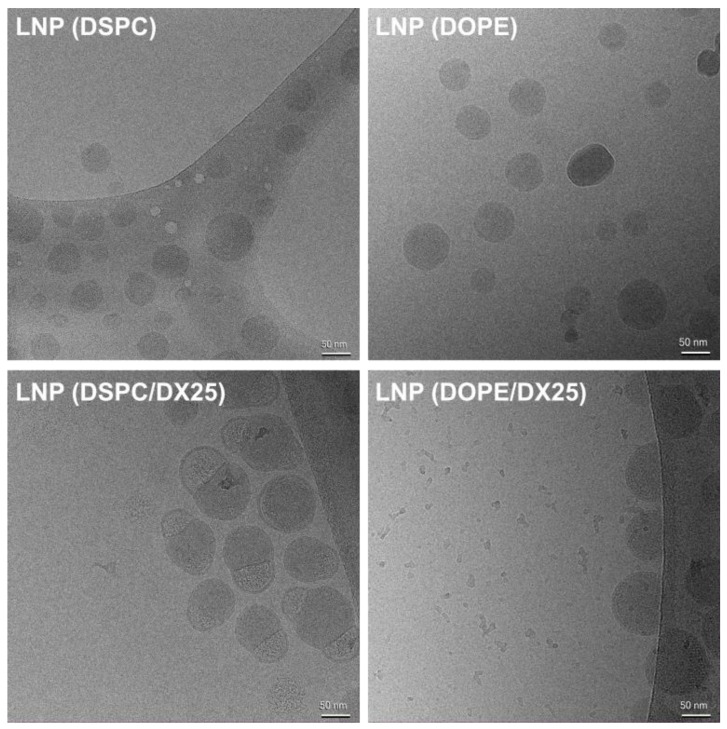
Cryo-TEM images of LNPs composed of DSPC, DOPE, DSPC/DX25, and DOPE/DX25.

The presence and morphology of stable lipid nanoparticles (LNPs) were studied using cryo-TEM analysis (Figure 4).

DSPC- and DOPE-containing LNPs without DX exhibited spherical-shaped nanoparticles with regular size and shape, ranging from 50 to 70 nm. In agreement with previous reports, LNP (DSPC) displayed bilamellar round particles with no internal defects and occasionally exhibited characteristic “blebs” commonly associated with formulations transporting large nucleic acids, such as mRNA [6]. These “blebs” are mostly absent in LNP(DOPE). DOPE, unlike DSPC, can form electron-dense non-bilayer structures even when excluded from mRNA-ionizable lipid structures.

In the case of DX-loaded LNPs, nanoparticles in the range of 50 to 70 nm were also observed. However, for LNP(DSPC)/DX, the morphology tended to resemble spheres with surface bubbles (blebs), sometimes with more than one. In contrast, LNP(DOPE)/DX showed a more conserved morphology, with nanoparticles appearing more like the unloaded LNP(DOPE).

**Figure 5:**
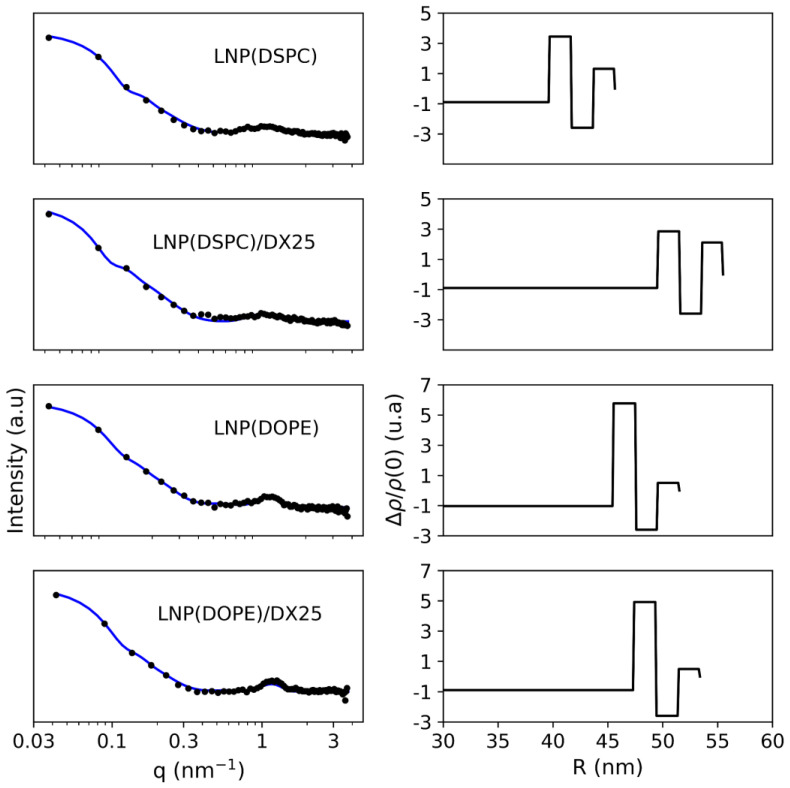
SAXS profiles of the different LNP and LNP/DX formulations. The left column shows the Loglog plot of the experimental SAXS pattern in (symbol) and the fitted curve in continuous line. The right column shows the electron density profile obtained from the bilayer model in function of the radius of the particle. The density distribution is affected by the particle surface composition.

The crystallographic structure of LNPs was studied using SAXS (Figure 5). The SAXS patterns suggest the presence of spherical nanoparticles, potentially bilamellar vesicles or lipidic nanoparticles, displaying a distinctive bump centered near 1 nm^−1^. This signal primarily could be attributed to the size of the nanoparticles and lipid stacking within them. The mean nanoparticle size derived from the small-angle region is documented in Table 1 with a fixed polydispersity of 15%. While this polydispersity exceeds the PDI registered by dynamic light scattering (DLS), the simulated intensity does not account for smearing effects or surface defects that might smooth the pattern, as they mainly influence oscillations rather than the overall signal. During the least squares fitting routine, only the contributions from the polar heads were considered. The electron density of the solvent was set to 0, while the core of the lipid nanoparticle and the low-density region of the bilayer were set to −1 and −2.5, respectively. This simplification aimed to reduce the number of variables to be fitted and decrease the correlation between non-linear variables.

The analysis revealed differences in the surface structure determined by the bilayer between DSPC and DOPE, with DOPE exhibiting a higher electron density in the inner polar shell and more stacked layers compared to DSPC. Despite minimal effects on the electron density profile due to DX, an increase in the mean particle diameter was observed for both DSPC and DOPE LNPs (Table 1). This finding suggests that DX was mostly incorporated within the LNPs, as the number of multilayers did not show any changes.

**Figure 6:**
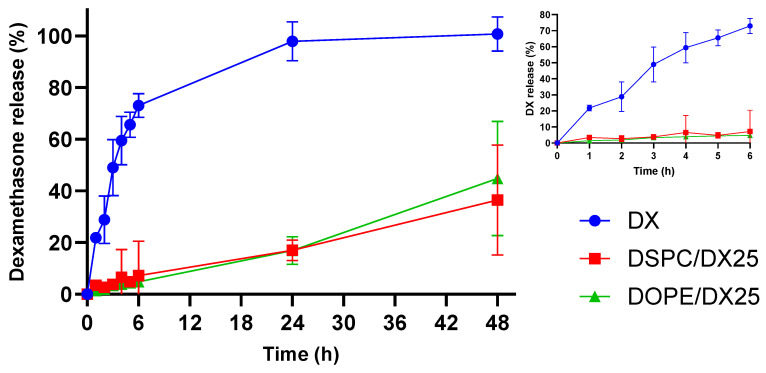
In vitro DX release from LNPs composed of DSPC and DOPE followed for 48 h. Release was performed in PBS 10 mM at pH = 7.4 and 37 °C. The kinetics were compared with the diffusion of free DX across the dialysis device. The inset shows the DX release at the first 6 h. Results are expressed as mean ± SD (n = 3).

Furthermore, in vitro DX release from LNPs in PBS was studied to determine the pharmacokinetics after encapsulation (Figure 6). First, free-soluble DX was tested as a control. During the first 6 h, free DX diffused across the dialysis device, with 80% of the initial content reaching the release medium. This slow diffusion could be attributed to the low solubility of DX in physiological environments. On the other hand, DX encapsulated in LNPs showed a slower release, reaching values close to 5% during the first 6 h for LNPs containing either DSPC or DOPE. This finding suggests that DX was, most likely, incorporated into the core of the nanoparticles. After 24 h, a 20% release of DX was observed from the LNP formulations, while 100% of the free DX control had completely diffused into the release medium. Longer incubation times demonstrated the ability of LNPs to release DX in a continuous and sustained manner. Regarding the stability of the formulations, the release profile remained unchanged after 1 month of storage, indicating no leakage of DX over time (Figure S2).

**Figure 7:**
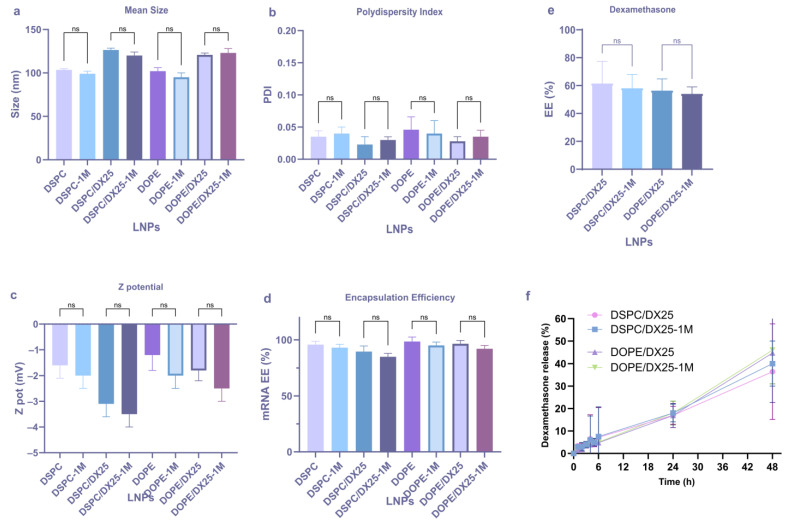
Physical stability of DX-LNPs. LNPs demonstrated stability for at least one month (1M) when stored at 4 °C and protected from light, with no significant changes observed in size (**a**), PDI (**b**), Z pot (**c**), and mRNA EE% (**d**). Additionally, the DX-loaded LNPs showed great stability in terms of DX EE (**e**) and maintaining the DX release (**f**). The results represent the mean (n = 3) ± SD. One-way ANOVA followed by Fisher’s LSD test was used to compare among groups: ns (not significant; *p* > 0.05).

Finally, the physical stability of the formulations was determined (Figure 7). The LNPs demonstrated stability for at least one month when stored at 4 °C and protected from light, with no significant changes observed in size, PDI, Z pot, and mRNA EE (*p* > 0.05). In addition, the DX-loaded LNPs showed great stability in terms of DX EE and maintaining the DX kinetic release, indicating the absence of leakage during storage.

### 2.4. In Vitro mRNA Transfection Efficiency of DX-Loaded LNPs

**Figure 8:**
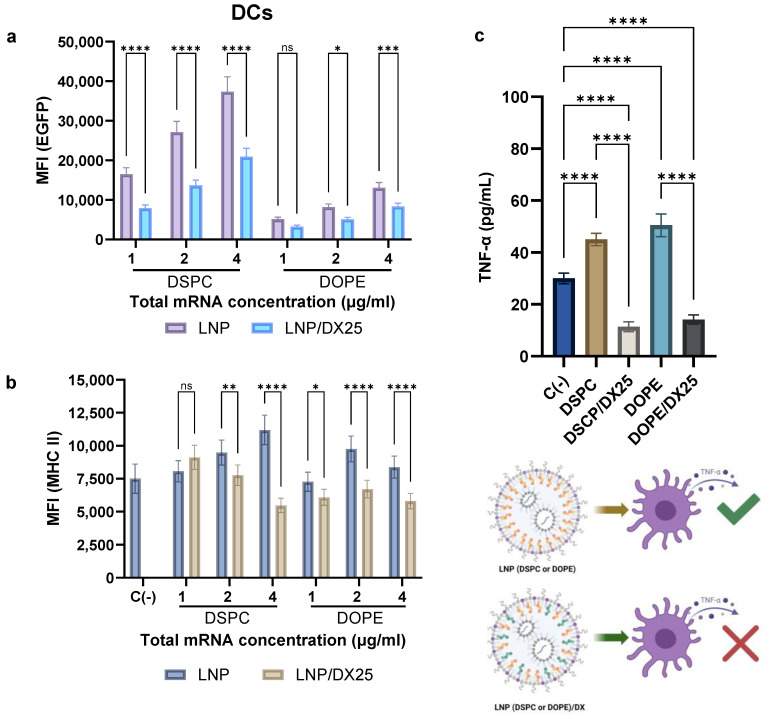
mRNA transfection (**a**) and MHC II expression (**b**) in dendritic cells (DC2.4) after incubation with LNPs and DX/LNPs at increasing concentrations of mRNA (1, 2, and 4 µg/mL of total mRNA) for 24 h. Mean fluorescence intensity (MFI) was analyzed by FACS. TNF-α cytokine release was also determined for all LNP formulations, suggesting that DX-loaded LNPs did not induce cytokine release, as depicted in the scheme (**c**). Results represent the mean (n = 3) ± SD. One-way ANOVA followed by Fisher’s LSD test was used to compare between groups: *p* < 0.05 (*), *p* < 0.01 (**), *p* < 0.001 (***), *p* < 0.0001 (****), and ns (not significant; *p* > 0.05).

The mRNA translation efficiency was tested in DC 2.4 cells at increasing doses of total mRNA, with the concomitant increase in DX dose, for the DX-LNP formulations containing either DOPE or DSPC (Figure 8). The expression of EGFP was assessed, with 100% of the cells being effectively transfected. Based on the mean fluorescence intensity per cell, an increase in mRNA expression was observed as the amount of LNP (and total mRNA) increased for all formulations. A significantly higher GFP expression was observed for the DSPC formulation compared to DOPE, with values approximately four times higher for the DSPC LNPs. On the other hand, a half decrease in expression was observed in the DX-loaded LNPs compared to their respective unloaded LNPs (Figure 8a).

To further evaluate the immunogenicity of LNPs, the expression of activation markers was assessed (Figure 8b). The presence of the MHC class II molecule, which is relevant in the antigen presentation process, was constitutively expressed in the dendritic cells, and the total population of DCs expressing this cell marker was not affected by the presence of LNP or LNP/DX. However, a significant decrease in the expression of MHC II molecules on the DC surface was observed after treatment with DX-loaded LNPs. A 30% to 50% reduction was observed at the maximum dose for both DSPC and DOPE LNPs. Moreover, stimulation of DCs by LNPs resulted in a significant release of TNF-α compared to non-treated cells (Figure 8c). However, after treatment with DSPC and DOPE LNPs loaded with DX, a decrease in TNF-α concentration of approximately fourfold was observed, with levels falling below the baseline of non-treated cells.

**Figure 9:**
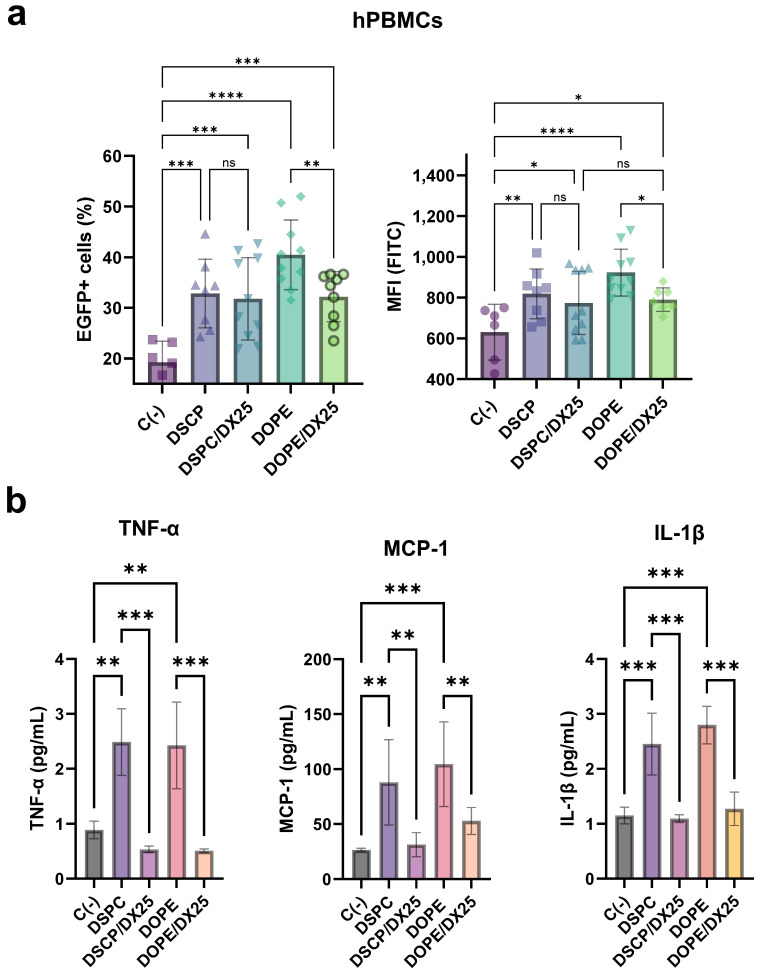
Transfection of hPBMCs with EGFP mRNA-loaded LNPs. The resulting percentage of EGFP+ cells and the mean fluorescence intensity (MFI) are shown (**a**). The cytokine release of TNF-α, MCP-1, and IL-1β was measured in the supernatants of stimulated hPBMCs with 8 µg/mL of mRNA loaded into LNPs (**b**). The results represent the mean (n = 3) ± SD. One-way ANOVA followed by Fisher’s LSD test was used to compare among groups: *p* < 0.05 (*), *p* < 0.01 (**), *p* < 0.001 (***), *p* < 0.0001 (****), and ns (not significant; *p* > 0.05).

The transfection efficiency of LNP/DX formulations was also evaluated in human hPBMC cultures by FACS analysis (Figure 9 and Figure S2).

hPBMCs are a diverse mixture of immune cells, making them a relevant target cell population to evaluate the immunosuppressive effects of LNP-formulated DX. Analysis with mRNA-encoded EGFP showed that all formulations were able to transfect cells in the range of 30% to 40% (Figure 9a). There was no significant difference between LNP(DSPC) and LNP/DX(DSPC) in the percentage of transfected cells and mean EGFP expression per cell. In the case of LNP(DOPE), the incorporation of DX into LNP resulted in a slight decrease in transfection efficiency, but still with expression levels comparable to LNP/DX(DSPC). EGFP expression was statistically significant (*p* < 0.05) compared to the negative control.

Immune stimulation of hPBMCs by LNP formulations induced the secretion of inflammatory cytokines and chemokines, such as TNF-α, MCP-1, and IL-1β (Figure 9b). Regardless of the presence of DSPC or DOPE, TNF-α levels increased approximately 2.5-fold compared to untreated hPBMCs. Meanwhile, a 5-fold reduction of each cytokine and chemokine measured was observed after treatment with DX-loaded LNPs. Similarly, the LNP formulations exhibited a 3- to 4-fold increase in the chemokine MCP-1, a cytokine related to the inflammatory process that attracts and enhances the expression of other inflammatory factors and cells [18]. A reduction in MCP-1 levels by about two times was observed with the treatment of DX-LNPs. IL-1β, a potent pro-inflammatory cytokine involved in host defense against infection and injuries, was also detected after stimulation [19]. An approximately 2.5- to 3-fold increase was observed for the LNP-based formulations, while the same reduction rate was produced with DX-loaded LNPs. These results confirmed that treatment with DX-loaded LNPs reduced the levels of these cytokines and chemokines, suggesting that DX acts as an immunoregulator even when integrated into the LNP structure.

### 2.5. Biocompatibility of DX-Loaded LNPs

**Figure 10:**
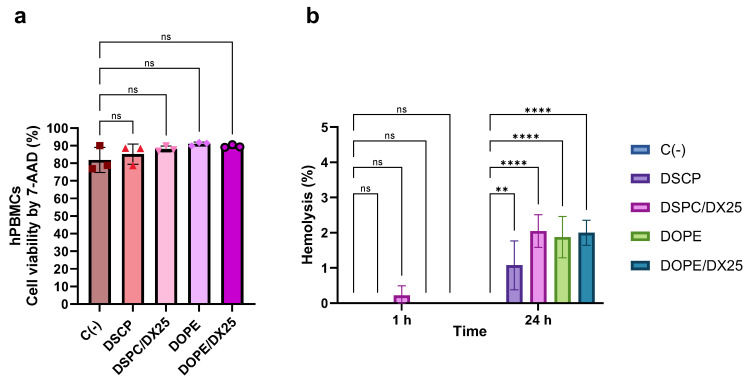
Biocompatibility of DX-loaded LNPs. The cell viability of hPBMCs was determined by incorporation of the 7-AAD with flow cytometry (**a**). The hemotoxicity of the different LNP formulations was also assessed (**b**). The results represent the mean (n = 3) ± SD. One-way ANOVA followed by Fisher’s LSD test was used to compare among groups: *p* < 0.01 (**), *p* < 0.0001 (****), and ns (not significant; *p* > 0.05).

Cell viability of hPBMCs treated with the different LNPs was determined using the fluorescent intercalant dye 7-Aminoactinomycin D (7-AAD). This dye is excluded from cells with intact membranes but can enter into damaged cells (Figure 10a). It was observed that hPBMCs transfected with both empty and DX-loaded LNPs showed no significant changes in cell viability (*p* ≥ 0.05). Additionally, the hemotoxicity of the formulations was evaluated to determine their biocompatibility (Figure 10b). The interaction of LNPs with erythrocytes is a relevant method to assess their safety. According to the ISO/TR 7406 standard, biomaterials with less than 5% hemolysis are considered safe for biomedical applications. After 1 h of exposure to LNPs, no hemotoxicity was observed for any of the formulations tested. After 24 h, a minimal degree of hemolysis of less than 2% was observed in all formulations, indicating their biocompatibility for in vivo applications. The preservation of cell viability following treatment with DX-LNP provides further evidence that the inhibition of cytokine release can be attributed to the delivery of bioactive DX rather than to a loss of cell viability.

### 2.6. In Vivo Biodistribution

**Figure 11:**
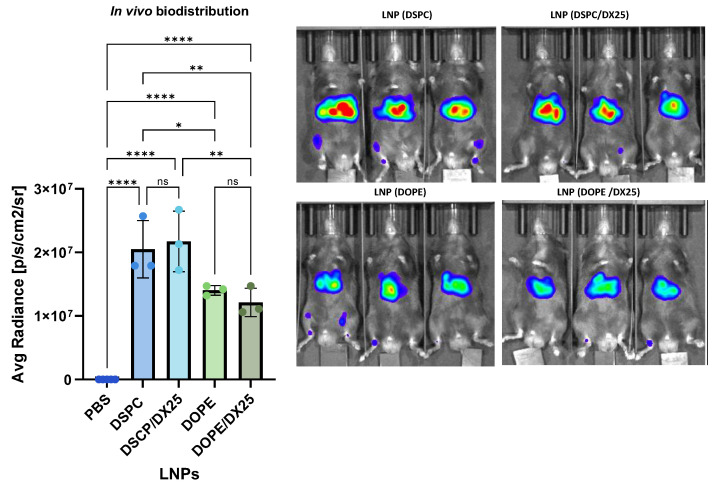
In vivo biodistribution of the LNPs determined in a C57BL/6 naïve mice after i.m. injection with the LNP formulations delivering Luc-mRNA. The graphs represent the mean of the region of interest (ROI) (n = 3) ± SD. One-way ANOVA followed by Fisher’s LSD test was used to compare between the groups. *p* < 0.05 (*), *p* < 0.01 (**), *p* < 0.0001 (****), and ns: not significant. Exposure time was 5 s.

The in vivo biodistribution of DX-loaded LNPs in a C57BL/6 naïve mice was assessed. The mice were intramuscularly injected with Luc mRNA-LNP at a dose of 7.0 µg per mouse. After 6 h, bioluminescence throughout the body was quantified (Figure 11). All LNP and DX-loaded LNP formulations exhibited Luc expression. There were no significant differences in Luc expression between the LNP and DX-loaded LNP formulations (*p* > 0.05), suggesting that DX does not affect in vivo expression. While the DSPC formulation exhibited an average radiance of approximately 2 × 10^7^, the DOPE formulations showed bioluminescence values that were 2-fold lower (*p* < 0.05).

**Figure 12:**
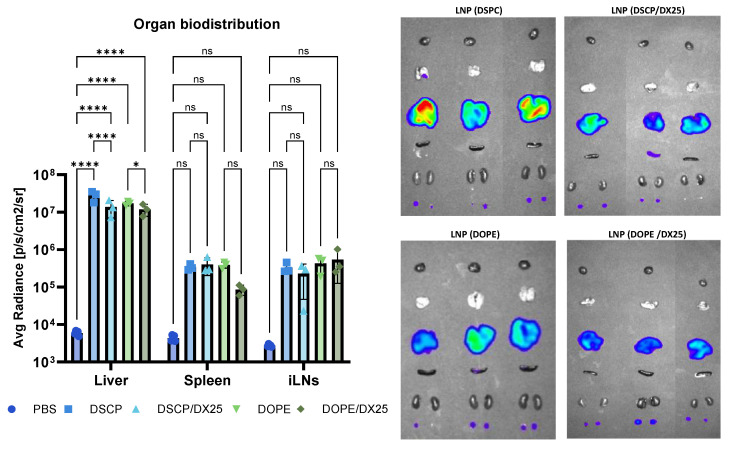
The in vivo biodistribution at organ levels of the LNPs in C57BL/6 naïve mice after i.m. injection with the LNP formulations delivering Luc-mRNA. The graphs represent the mean (n = 3) ± SD. Two-way ANOVA test was used to compare among groups. *p* < 0.05 (*), *p* < 0.0001 (****), and ns: not significant.

Regarding organ biodistribution, ex vivo imaging was also performed (Figure 12). The imaging of various tissues revealed the strongest signal in the liver. Although a lower Luc expression on the order of 5 × 10^5^ luminescent units was observed in the spleen and inguinal lymph nodes of mice treated with both LNP and DX-loaded LNPs, these values were not significantly different compared to liver values, which were nearly 100 times higher. These results suggest that almost all the DX-loaded LNPs target liver tissues, making them a promising tool for expressing target proteins and potentially reducing inflammatory effects associated with liver-related pathologies. The change in lipid composition from DSPC to DOPE did not appear to affect the final biodistribution of the DX-LNPs following intramuscular administration.

## 3. Discussion

The rapidly advancing field of mRNA therapeutics faces the critical challenge of balancing the broader goal of enhancing therapeutic efficacy while minimizing the side effects of the formulation [1]. A common component of LNPs is polyethylene glycol (PEG), which is well known to induce anti-PEG antibodies [4,5]. In the context of protein replacement therapies, side effects can be further potentiated due to allergic responses against the administered protein. This is particularly relevant for inborn errors of metabolism, which require lifelong and frequent replacement of the corresponding enzyme/protein.

Recently, we demonstrated the efficacy of mRNA-based treatment of tyrosinemia and phenylketonuria in a mouse model, underlining the enormous potential of this therapeutic approach [14,15]. Thus, the design of LNPs formulated with immunosuppressive drugs is a promising approach capable of avoiding sensitization against the expressed antigen and components of the LNP formulation [3]. Furthermore, the targeted delivery of immunosuppressive drugs can be exploited in the treatment of auto-inflammatory diseases affecting the liver and other organ systems.

The present study investigated the potential of DX-LNPs as a dual system for exerting anti-inflammatory properties while effectively delivering mRNA. The initial screening of LNPs with varying compositions and increasing DX concentrations, achieved through partial cholesterol replacement, demonstrated that a 25% cholesterol substitution with DX represented the most promising ratio for the intended effects.

Although DX and cholesterol are structurally related, both belonging to the steroid family, slight differences in their functional groups inherit the potential to induce distinct effects on the LNP structure. Similar approaches by Zhang et al. and Cheng et al. proposed cholesterol replacement with 10% and 20% DX as optimal for mRNA delivery [1,10]. However, release kinetics and encapsulation efficiency of DX within the LNPs remain to be elucidated, along with further evaluation of this formulation in human cells. Notably, human cells are (1) more sensitive to inflammation than those of mice, and (2) they also differ in their responsiveness to DX formulations [20].

In our study, we identified that a 25% replacement of cholesterol with DX served as the most optimal formulation and demonstrated that increasing DX concentrations not only affected mRNA encapsulation efficiency but also impacted mRNA expression after transfection. Importantly, we determined the actual DX load by calculating the encapsulation efficiency (approximately 60%), which was not reported in other studies. These effects could be attributed to differences in hydrophobicity; cholesterol has a logP of 7.02, while DX has a logP of 1.87, indicating that cholesterol is around 140,000 times more soluble in organic solvents than DX. Additionally, maintaining cholesterol’s role within the LNP architecture is crucial, as it significantly enhances LNP stability and the ability to fuse with membranes by regulating membrane integrity and rigidity [9].

We delineated to our knowledge for the first time how DX is integrated into the LNP structure. SAXS analysis revealed that DX is incorporated primarily into the LNP core. Furthermore, we observed that the encapsulation efficiency for DX was approximately 60% for both the DSPC and the DOPE. These findings indicate that LNPs are unable to accommodate the full range of DX initially added into the lipid phase. This suggests that the subtle variations in chemical structure associated with cholesterol may exert an influence on LNP integrity. Importantly, stable DX-containing LNPs were synthesized, which demonstrated the ability to transfect different cell types, including HepG2, DCs, and hPBMCs.

One of the major challenges associated with DX administration is the side effects associated with systemic administration [13]. Several studies have attempted to slow down the release of DX from nanoparticles. Among the different approaches, those involving DX incorporation into lipid nanocarriers achieved desirable DX release profiles but did not prevent a fast initial release [21]. In our approach, we demonstrated for the first time that negligible DX release occurs during the first hour, suggesting that DX is not just a cargo molecule but also an integral part of the LNP architecture. However, when LNP stability is compromised, DX release begins. Considering that the LNP circulation time is relatively short, specific organs can be targeted within seconds, thus avoiding systemic distribution and side effects of DX [22].

The reduction in mRNA expression observed in DX-LNPs, particularly in vitro, raised additional questions. It has been reported that DX can reduce cellular metabolism, potentially slowing down the process of mRNA translation [23]. Therefore, adjustment of the cholesterol-to-DX replacement ratio is necessary to avoid a significant reduction in expression. This observation was further supported by the study of activation markers in DCs. Since dendritic cells, B cells, and macrophages constitutively express MHC class II molecules and are considered “professional” antigen-presenting cells (APCs) of the immune system, it was observed that treatment with DX-LNPs led to a reduction in MHC II molecule expression on the cell surface. Despite DX being integrated into the core of the synthesized LNPs, DX-LNPs suppressed he secretion of pro-inflammatory cytokines from DCs and hPBMCs. In accordance, previous studies demonstrated that the release of TNF-α can be reduced with LNP-DX treatment in vivo.

Finally, it was observed that most of the DX-LNPs accumulated in the liver. These results align with findings by Cheng et al., who also reported similar biodistribution patterns [10]. Although some studies have suggested that DX could enhance mRNA expression by reducing inflammation, we observed no enhancement in expression levels; instead, the expression levels were comparable to those of non-DX-loaded LNPs [1]. This indicates that DX does not interfere with the translation of the reporter mRNA. Despite modifying the LNP composition by replacing DSPC with DOPE, the biodistribution did not change after intramuscular injection. Mice treated with both LNPs and DX-loaded LNPs primarily showed accumulation in the liver, with expression levels 100 times higher than in other organs. These findings are consistent with a study by Pateev et al., which demonstrated that after administering LNPs containing Luc mRNA, bioluminescence was primarily observed in the liver, with additional signals detected in the spleen [24]. This is in contrast to observations after intravenous injection, where DSPC-based LNPs typically target the spleen, and DOPE-based LNPs target the liver [17]. However, changes in physicochemical properties were observed with DOPE formulations, characterized by a more relaxed structure and the absence of blebs. DOPE, unlike DSPC, can still form electron-dense non-bilayer structures even when excluded from mRNA-ionizable lipid assemblies [6,25].

In summary, the outlined observations demonstrate the promising properties of LNPs composed of ALC-0315, DSPC/DOPE, cholesterol/DX, and ALC-0159 for improving mRNA delivery with low reactogenicity and reduced inflammatory response. The additional anti-inflammatory properties conferred by encapsulated DX offer new opportunities for targeted anti-inflammatory treatment of liver diseases. Additionally, the DX-LNPs represent a novel formulation that can be applied in enzyme replacement therapies, e.g., for inborn errors of metabolism affecting the liver, such as PKU or Tyrosinemia [14,15]. The required lifelong treatment with frequent dosing inherently carries the risk of sensitization against the delivered protein and LNP components, which can potentially be mitigated through the strategy presented herein.

## 4. Materials and Methods

### 4.1. Materials

Dexamethasone (DX) was purchased from Sigma-Aldrich (Buenos Aires, Argentina). The (4-hydroxybutyl)azanediyl)bis(hexane-6,1-diyl)bis(2-hexyldecanoate) (ALC-0315) and alpha-[2-(ditetradecylamino)-2-oxoethyl]-omega-methoxy-poly(oxy-1,2-ethanediyl) (ALC-0159) were purchased from Cayman Chemical (Ann Arbor, MI, USA). 1,2-distearyol-sn-glycero-3-phosphoethanolamine (DSPC), 1,2-Dioleoyl-sn-glycero-3-PE (DOPE), and other lipids were obtained from Avanti Polar Lipids (Alabaster, AL, USA). CleanCap^®^ Enhanced Green Fluorescent Protein (EGFP) mRNA and CleanCap^®^ Firefly Luciferase (Luc) mRNA were purchased from TriLink BioTechnologies (San Diego, CA, USA). Both mRNA constructs were nucleotide modified using 5-methoxyuridine to replace native uridine.

### 4.2. Synthesis of LNP Formulations

LNPs were synthesized by a self-assembly method combining an aqueous mRNA solution (either EGFP or Luc) at a pH of 4.0 in a 50 mM citrate buffer (Merck, Darmstadt, Germany) with a lipid-containing ethanolic phase using two microfluidic platforms, depending on the sample size required for in vitro/in vivo experiments.

#### 4.2.1. DX-Loaded LNPs with NanoAssemblr^®^ Spark™

As a first in vitro approach, LNPs were prepared by mixing an aqueous solution of the mRNA at 350 µg/mL with an ethanolic phase containing the lipids at 25.0 mM using the NanoAssemblr^®^ Spark™ (Precision NanoSystems Inc., Vancouver, BC, Canada). The ethanol (AppliChem, Darmstadt, Germany) and aqueous phases were mixed at an N/P ratio of 6 and flow rate ratio (FRR) of 2:1 (aqueous: organic). Setting 3 was selected for the preparation with load volumes of 48 µL, 48 µL, and 24 µL of PBS, mRNA phase, and lipid phase, respectively. The organic phase was prepared by means of changing the percentage of DX by substituting cholesterol. The exact composition and molar ratios for each lipid mix mixture are further discussed in the Results section. After synthesis, LNPs were diluted 1:20 (for in vitro) in 1X phosphate-buffered saline (PBS) and concentrated in Amicon^®^ Ultra-0.5 mL centrifugal filters of 10,000 MWCO (Merck, Darmstadt, Germany) at 1000× *g* for 10 min per fraction. The resulting solution was then stored at 4 °C for further use.

#### 4.2.2. DX-Loaded LNPs Composed of DSPC and DOPE Using the NanoAssemblr^®^ Ignite™

LNPs were formulated using an aqueous mRNA solution at a concentration of 120 µg/mL (EGFP mRNA or Luc mRNA), combined with a lipid-containing ethanolic phase at 12.5 mM, employing the NanoAssemblr^®^ system (Precision NanoSystems Inc., Vancouver, BC, Canada). The ethanol and aqueous phases were mixed at a total flow rate of 12 mL/min, with a N/P ratio of 6 and an FRR of 3:1 (aqueous to organic). Initial and final waste volumes were 200 µL and 50 µL, respectively. Various LNPs were synthesized by modifying the type of phosphatidylcholine used (DSPC or DOPE). The specific composition and molar ratios for each lipid mixture were detailed in the Section 2. Post-synthesis, the LNPs were diluted at ratios of 1:20 for in vitro and 1:40 for in vivo use in 1X PBS, then concentrated using Amicon^®^ centrifugal filters of 50,000 MWCO(Merck, Darmstadt, Germany) at 2000× *g* for 5 min per fraction. The resulting solution was filtered through a 0.22 µm filter and stored at 4 °C for future applications.

### 4.3. Determination of mRNA Encapsulation Efficiency (EE) by Ribogreen Assay

The mRNA was quantified by fluorescence intensity using Ribogreen reagent (Thermo-Fisher, Waltham, MA, USA) at an emission wavelength of 535 nm and an excitation wavelength of 485 nm, utilizing a TECAN Spark^®^ plate reader (Männedorf, Switzerland). To determine mRNA encapsulation efficiency, the mRNA concentration in the LNP samples was measured under two conditions: with and without incubation in 2% Triton X-100 (Merck, Darmstadt, Germany) at 37 °C for 10 min. These conditions measured total mRNA and unencapsulated mRNA, respectively. The encapsulated mRNA was calculated by subtracting the unencapsulated mRNA value from the total mRNA value.

### 4.4. Particle Size, Zeta Potential (Z Pot), and Polydispersity Index (PDI)

Measurements were conducted on 1/100 PBS dilutions of each formulation. The average hydrodynamic diameter and size distribution (PDI) of the LNP formulations were assessed in triplicate using dynamic light scattering (DLS) on a Nano ZS Zetasizer (Malvern Instruments Corp., Malvern, UK) in ZEN0040 disposable cells (Brand, Wertheim, Germany). Zeta potentials (Z pot) of the various LNP formulations were measured with the same instrument using DTS1080 disposable capillary cells (Malvern Instruments Corp., Malvern, UK). The stability of the formulations was monitored for changes in particle size, Z pot, and encapsulation efficiency (EE) after storage at 4 °C for up to one month.

### 4.5. Cryogenic Transmission Electron Microscopy (Cryo-TEM)

The size, morphology, and distribution of the LNPs were verified by cryo-TEM. For cryo-TEM analysis, samples were vitrified with a Vitrobot Mark V (ThermoFisher, Hillsboro, OR, USA). A 3 µL sample dispersion was applied to a Quantifoil or lacey carbon-coated TEM grid that had been glow-discharged in an oxygen plasma cleaner (Diener Nano^®^, Diener electronic, Ebhausen, Germany) shortly beforehand. After removing the excess solution with filter paper, the grid was rapidly immersed in liquid ethane. The sample was then transferred to a TEM (FEI Titan Krios G4, Thermo Fisher Scientific, Naarden, The Netherlands) under cryogenic conditions. Conventional TEM imaging was conducted with an acceleration voltage of 300 kV. Micrographs were captured using a 4k Direct Electron Detection Camera (Gatan K3, Pleasanton, CA, USA) under low-dose conditions. The images were subsequently analyzed using ImageJ^®^ Software (version 1.54).

### 4.6. Small-Angle X-Ray Scattering (SAXS)

The SAXS profiles were acquired at the NCD-SWEET beamline (Project ID 2023067620) at the ALBA Synchrotron Light source in Barcelona, Spain. The incoming energy was set at 10 keV, with a sample-to-detector distance of 3.2 m. Liquid samples were placed in low-scattering polymeric capillaries with an external diameter of 2.2 mm and a wall thickness of 0.1 mm. Two-dimensional patterns were captured using a Pilatus 1M detector (Dectris, Baden, Switzerland), and one-dimensional patterns were derived through azimuthal integration using the pyFAI library [26]. The intensity was expressed as a function of the scattering momentum transfer *q* ((q=4π/λ sin(θ)), which is dependent on the incoming wavelength (*λ*) and the scattering angle (2 θ). For each sample, 10 frames of 10 s were recorded, with potential radiation damage to the samples being discarded. The measurements were performed at room temperature (22 °C).

To investigate the structural difference between formulations, a polydisperse multi-shell spherical particle model was used to account for the mean electron density difference from the outer shells of each product [27]. The form factor (*P*) of a single particle was expressed according to the following equation:

$$P(q,R)={\left[\rho_{1}A(R_{1},q)+\sum_{i=2}^{N}\left(\rho_{i}-\rho_{i-1}\right)A(R_{i},q)\right]}^{2}/\;{\left[\rho_{1}V(R_{1})+\sum_{i=2}^{N}(\rho_{i}-\rho_{i-1})V(R_{i})\right]}^{2}$$

where *A* is the volume weighted amplitude of a homogeneous particle of radius *Ri*

$$A(R_{i},q)=4\pi \frac{sin(Rq)-Rqcos(Rq)}{q³}$$

*V* is the volume of a sphere (4π/3 R³), and $\rho_{i}$ is the average electron density of region *i* of the particle. In our current model, we adapted a strategy like that used by other researchers, who estimated the radial distribution scattering density for neutrons in a multi-shell particle [28]. For this study, we considered a bilayer nanoparticle with a homogeneous core. These particles are characterized by an average radius (*R_av_*) with a standard deviation (*σ*) set at 20% using a Gaussian distribution function (*D*). For the bilayer outer shell, we included three contributions: two for the high-density polar regions and one for the low electron-density region. The thickness of each shell was fixed at 2 nm, reflecting the nature of the lipids and their components. The total scattering intensity can be expressed with the following equation:

$$I\;\left(q\right)=\left(cte_{1}+cte_{2}S_{eff}\left(q\right)\right)\int_{0}^{\infty }D\left(R,R_{av},\;\sigma \right)P\left(q,R,\;\vec{\rho }\right)dRback$$

where *S_eff_* is the structure factor of a lamellar system (multilamellar). A para-crystal structure factor was employed [29]. The disorder parameter for the repeat distance was set to 0.01. Under these constraints, the variables included the mean core radius, *R_av_*, the electron density of the concentric shells, and the average number of layers.

### 4.7. In Vitro Release Studies of DX from LNPs

The analytical protocol for detecting DX concentration was initially established at λ_max_ = 242 nm using a UV–Vis TECAN Spark^®^ plate reader (Männedorf, Switzerland). A linear relationship was observed within the range of 0.25 to 30 µg/mL (r^2^ = 0.99). The in vitro drug release assay was conducted using Slide-A-Lyzer^®^ MINI dialysis devices MWCO 10 kD (ThermoScientific, Rockford, IL, USA). Each device was filled with 500 µL of the respective formulation and immersed in 3.5 mL of PBS at pH 7.4 and 37 °C, with continuous shaking at 100 rpm [21]. For the free drug release study, a stock solution of 80 µg/mL was prepared in PBS, and 0.5 mL was transferred to the release device. Samples of 200 μL were taken at regular intervals over a period of 48 h, and the DX concentration was measured by UV–Vis spectroscopy at λ_max_ = 242 nm. An additional 200 μL of fresh medium was added to maintain a constant volume.

### 4.8. Evaluation of Transfection Efficiency In Vitro

The expression of the reporter EGFP-encoding mRNA encapsulated within different LNP formulations was assessed using a human hepatocellular carcinoma cell line (HepG2) and a mouse dendritic cell line (DC2.4). HepG2 cells were grown in RPMI 1640 medium supplemented with 10% heat-inactivated fetal bovine serum (iFBS), 1% antibiotics (100 U/mL penicillin and 100 μg/mL streptomycin), 1% (2mM) L-glutamine, and 5 mM HEPES. The cells were maintained at 37 °C in a humidified atmosphere containing 5% CO_2_. They were seeded in 24-well culture plates at a density of 2.5 × 10^5^ cells per well and allowed to grow for 24 h. Transfection with LNPs was performed using 500 ng of total mRNA per well (1 µg/mL) for 24 h. Post-incubation, green fluorescent cells were detected using an inverted fluorescent microscope (Olympus CKX41, Tokyo, Japan). The supernatant and cells (washed with PBS and treated with trypsin) were collected and washed with 2 mL fluorescence-activated cell sorting (FACS) buffer (1X PBS with 2% iFBS) to neutralize trypsin (TrpLE™ Express 1X, Gibco, ThermoFisher, Waltham, MA, USA). Following centrifugation at 400× *g* at 4 °C for 10 min, the cells were resuspended in 70 µL of FACS buffer and stained with 3 µL of 7-Amino-Actinomycin D (7-AAD) to assess viability. Samples were acquired using a flow cytometer (LSR II, BD Biosciences, Bedford, MA, USA), and data were analyzed with FlowJo™ software version 10.8 (Ashland, OR, USA; Becton, Dickinson and Company).

DC2.4 cells were cultured in RPMI-1640 medium supplemented with 10% heat-inactivated fetal bovine serum (iFBS), 100 U/mL penicillin, 100 µg/mL streptomycin, 2 mM L-glutamine, 1X non-essential amino acids, 1 mM HEPES, and 0.0054X β-mercaptoethanol. They were seeded in 24-well culture plates at a density of 4 × 10^4^ cells per well and incubated for 24 h. The cells were then treated with LNPs at a dose of 500 ng of total mRNA per well (1 µg/mL) for 24 h. For TNF-α analysis, 10 µL of the supernatant was collected. The same transfection protocol described for HepG2 cells was applied to DCs to determine transfection efficiency. As a control, mRNA premixed with Lipofectamine™ MessengerMAX Reagent (Invitrogen, Carlsbad, CA, USA) was used according to the manufacturer’s instructions.

### 4.9. Isolation, Seeding, and LNP Treatment of hPBMCs

Buffy coats were collected from healthy volunteers at the University Medical Center Mainz Blood Bank after obtaining informed consent. Following collection, 50 mL of blood was transferred to sterile flasks and diluted with 100 mL of Hank’s balanced salt solution (HBSS). Human peripheral blood mononuclear cells (hPBMCs) were isolated through density gradient centrifugation using Histopaque^®^ (Merck, Darmstadt, Germany) at room temperature. Specifically, 35 mL of the diluted blood was carefully layered over 15 mL of Histopaque^®^ and centrifuged at 900× *g* for 20 min at room temperature without applying a brake. The hPBMC layer was then carefully collected, washed with 50 mL of cold HBSS, and centrifuged at 400× *g* for 10 min at 4 °C. This washing step was repeated twice. The final pellet was resuspended in 50 mL of X-vivo medium (Lonza, Walkersville, MD, USA), supplemented with 100 U/mL penicillin and 100 µg/mL streptomycin. For cell counting, trypan blue was used.

For LNP transfection, hPBMCs were suspended in an X-vivo 15 medium (Lonza, Walkersville, MD, USA), supplemented with 100 U/mL penicillin and 100 µg/mL streptomycin. The cells were then plated in 48-well culture plates at a density of 2.5 × 10^5^ cells per well and incubated for 24 h. After this incubation period, cells were treated with various LNP formulations containing EGFP mRNA (8 µg/mL of mRNA each), along with appropriate controls. To enhance transfection, Apolipoprotein E3 was added at a concentration of 1.0 µg/mL, as identified in previous studies [6]. Following an additional 24-hour incubation, 30 µL of the supernatant was collected for cytokine analysis. To evaluate EGFP expression, cells were detached by incubation with 50 µL of trypsin at 37 °C for 10 min, followed by washing with FACS buffer and analysis by flow cytometry.

### 4.10. Cytokine Release Measurement

After treatment with LNP, the cell culture supernatants were collected and stored at −20 °C prior to cytokine measurements. TNF-α, CCL2 (MCP-1), CXCL8 (IL-8), IL-1β, IFN-γ, IL-6, CXCL10 (IP-10), and IL-4 levels were quantified using multiplex Cytometric Bead Assays (CBA): LEGENDplex™ HU Essential Immune Response Panel for hPBMC supernatants and LEGENDplex™ MU Th Cytokine Panel for DC2.4 cell supernatants (BioLegend, San Diego, CA, USA). Assays were performed according to the manufacturer’s protocol, and data acquired on an LSR II flow cytometer (BD Biosciences) were analyzed using the online LEGENDplexTM data analysis software version 8.0 (https://legendplex.qognit.com/workflow (accessed on 20 December 2023 and 13 February 2024).

### 4.11. Hemotoxicity Studies

Venous blood from healthy donors was provided by the Blood Bank of the University Medical Center Mainz after informed consent was obtained. The heparinized blood was aliquoted into 24-well culture plates and cultured in an X-vivo medium supplemented with 1% antibiotics. The blood was then exposed to 20 µL of each LNP formulation at 37 °C for either 1 or 24 h. Following incubation, the blood–LNP mixtures were centrifuged at 1200× *g* for 5 min at 37 °C. The precipitate was removed, and the degree of erythrocyte lysis was assessed by measuring hemoglobin release at λ = 540 nm in a TECAN Spark^®^ plate reader (Männedorf, Switzerland). To establish a baseline for complete hemolysis (100%), erythrocytes were treated with 1.0% Triton X-100, while the negative control consisted of erythrocytes incubated in PBS.

### 4.12. In Vivo Biodistribution Assay

All animal procedures described in this study were performed with the approval of the local authorities, specifically the Landesuntersuchungsamt Rhineland-Palatinate (reference number AK G 19-1-080).

C57BL/6 naïve mice received intramuscular injections (i.m.) in both tibialis anterior muscles with 50 µL (25 µL each) of Luc mRNA-loaded LNPs at a dose of 7.0 µg mRNA per mouse. Six hours after injection and 10 min before image acquisition, 150 µL of sterile-filtered luciferin substrate (20 g/L) (IVISBriteTM D-luciferin potassium salt) dissolved in PBS was administered intraperitoneally. Relative luciferase activity was evaluated in vivo in isoflurane–oxygen anesthetized mice using an IVIS Spectrum CT (PerkinElmer, Waltham, MA, USA).

Following imaging, mice were euthanized by cervical dislocation, and organs (heart, lungs, liver, spleen, kidneys, and inguinal lymph nodes) were dissected for ex vivo imaging. Organs were then weighed, and images were analyzed using Living Image^®^ Software version 4.7 (Caliper Life Sciences, Hopkinton, MA, USA).

### 4.13. Statistical Analysis

One-way ANOVA followed by Fisher’s LSD test was used to compare groups. For groups with multiple variables, the two-way ANOVA was selected. The *p*-values < 0.05 (*), *p* < 0.01 (**), *p* < 0.001 (***), and *p* < 0.0001 (****) were considered for significant differences. Non-significant differences are indicated as “ns”.

## Figures and Tables

**Table 1:** Mean diameter and fitting parameters to the multilayer model of the LNPs determined by SAXS.

Sample	Diameter (nm)	Multilayer (%)	N_av	*X* ^2^
LNP(DSPC)	91.8 ± 0.1	55.9	1.3 ± 0.3	0.77
LNP(DSPC)/DX25	111.0 ± 0.2	66.72	1.4 ± 0.3	1.31
LNP(DOPE)	103.0 ± 0.2	96.3	2.57± 0.01	0.53
LNP(DOPE)/DX25	106.8 ± 0.2	99.0	2.58 ± 0.01	1.05

## Data Availability

The original contributions presented in the study are included in the article/Supplementary Materials; further inquiries can be directed to the corresponding authors.

## Supplementary Materials

The following supporting information can be downloaded at: https://www.mdpi.com/article/10.3390/ijms252011254/s1.
