# Supplementary material for: Encapsulation of Dexamethasone into mRNA–Lipid Nanoparticles Is a Promising Approach for the Development of Liver-Targeted Anti-Inflammatory Therapies
Source: Int J Mol Sci. 2024 Oct 19;25(20):11254. doi: 10.3390/ijms252011254 (PMC11508592; doi:10.3390/ijms252011254)
Supplement: Supplementary file 1 [file ijms-25-11254-s001.zip › ijms-3247527-supplementary.pdf]

Supplementary

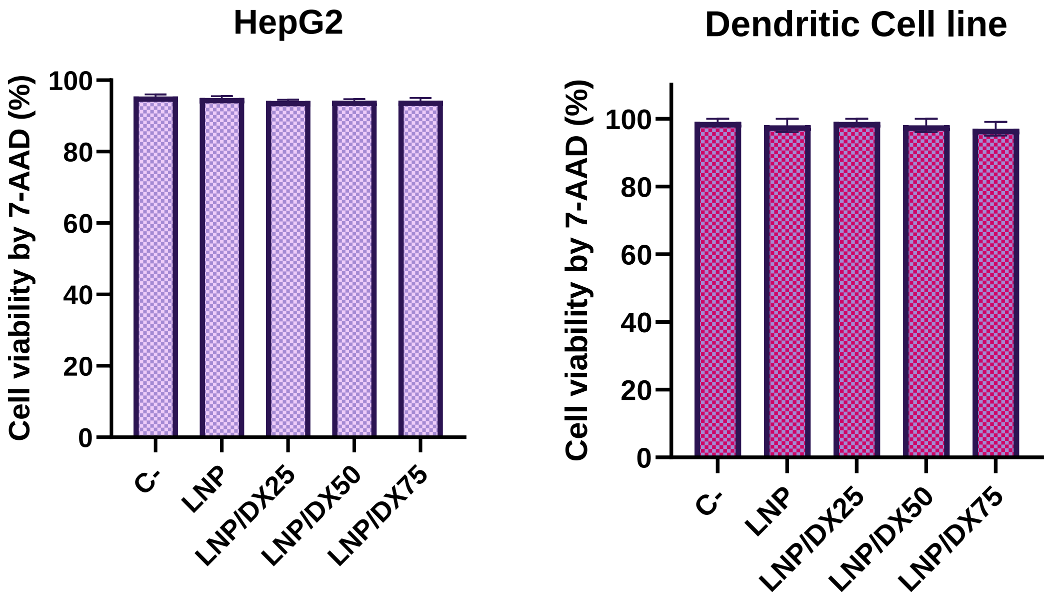

Figure S1 Cell viability of HepG2 and DC after LNP treatment determined by 7-AAD by FACS.

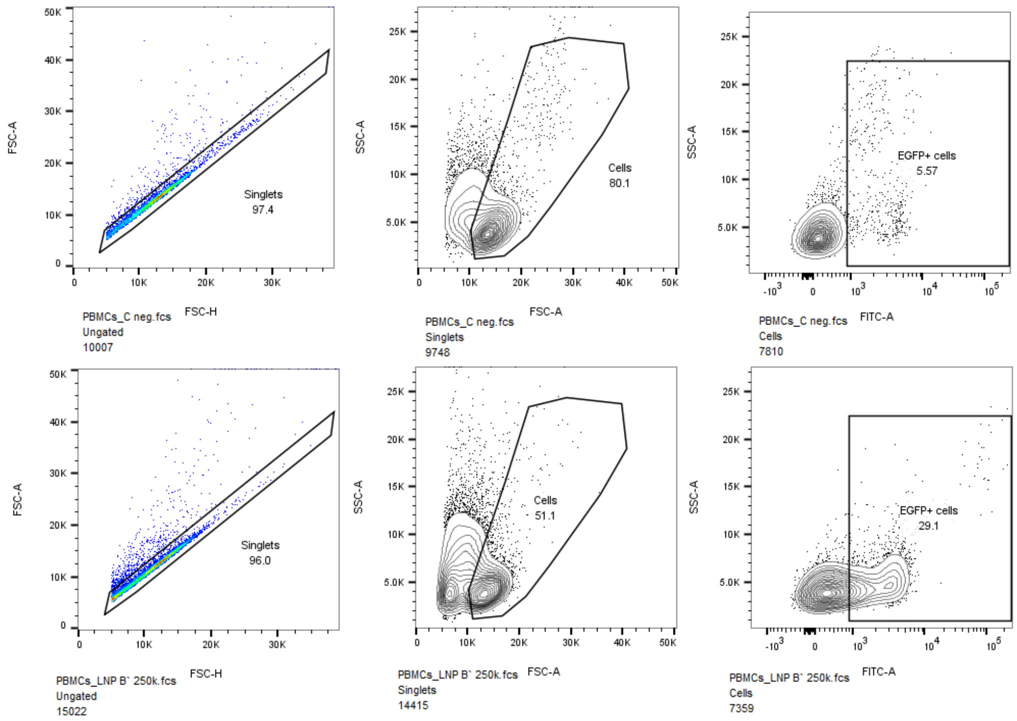

Figure S2 The gating strategy to determine the EGFP+ cells in human PBMCs transfected with LNPs by FACS analysis.
